# Supplementary material for: Performance assessment of high-density diffuse optical topography regarding source-detector array topology
Source: PLoS One. 2020 Mar 24;15(3):e0230206. doi: 10.1371/journal.pone.0230206 (PMC7092988; doi:10.1371/journal.pone.0230206)
Supplement: S1 Appendix — (DOCX) [file pone.0230206.s001.docx]

**Appendix I:**

The $\Delta OD$ is a function of $\Delta\mu_{a}\left( \lambda\right)$ and the relationship between $\Delta R$and the $\Delta OD$ has given by:

$$\log\left( \frac{R\left( \tau+T_{s} \right)}{R\left( \tau\right)} \right)=\Delta OD , \tau=nT_{s} ;n\in N=\{1,2,3,\ldots\} (1)$$

The reflectance due to absorption is a function of absorption coefficient [16]. Normalized first order approximation of the reflectance regarding individual channel can be written by:

$${{\Delta R}_{j}}^{pert}\left( \rho_{j},T_{s} \right)/ {R_{i}}^{0}\left( \rho_{j} \right)=\sum_{i=1}^{9} \Delta R^{a}\left( \rho_{j},T_{s},i \right) / {R_{i}}^{0}\left( \rho_{j} \right) (2)$$

Where,

$${R_{i}}^{0}\left( \rho_{j} \right)=\int_{0}^{+\infty} R_{i}\left( \rho_{j},t \right)dt=$$

$$-\frac{1}{4\pi}\sum_{m=-\infty}^{\infty} \left( \begin{aligned} z_{3m}\left( {\rho_{j}}^{2}+{z3m}^{2} \right)^{-\frac{3}{2}}\times\left\{ 1+\left[ \frac{\mu_{a}\left( {\rho_{j}}^{2}+z_{3m}^{2} \right)}{D} \right]^{\frac{1}{2}} \right\} \\ \times exp\left\{ -\left[ \frac{\mu_{a}\left( {\rho_{j}}^{2}+z_{3m}^{2} \right)}{D} \right]^{\frac{1}{2}} \right\}-z_{4m}{{(\rho_{j}}^{2}+z_{4m}^{2})}^{-\frac{3}{2}} \\ \times\left\{ 1+\left[ \frac{\mu_{a}\left( {\rho_{j}}^{2}+z_{4m}^{2} \right)}{D} \right]^{\frac{1}{2}} \right\}\times exp\left\{ -\left[ \frac{\mu_{a}\left( {\rho_{j}}^{2}+z_{4m}^{2} \right)}{D} \right]^{\frac{1}{2}} \right\} \end{aligned} \right) (3)$$

$${\Delta R}^{a}\left( \rho_{j},T_{s},i \right)=-\frac{1}{\left( 4\pi\right)^{2}D}\times\sum_{m=-\infty}^{\infty} \sum_{\eta=-\infty}^{\infty} \int_{Vi} d^{3}\vec{r_{i}}\delta\mu_{a}\left( \vec{r_{i}},T_{s} \right)\times\left( z_{23\eta}^{+^{'}}\frac{1+\mu_{eff}\rho_{23\eta}^{+^{'}}}{\left( \rho_{23\eta}^{+^{'}} \right)^{3}}\left\{ \frac{exp\left[ -\mu_{eff}\left( \rho_{12m}^{+}+\rho_{23\eta}^{+^{'}} \right) \right]}{\rho_{12m}^{+}}-\frac{exp\left[ -\mu_{eff}\left( \rho_{12m}^{-}+\rho_{23\eta}^{+^{'}} \right) \right]}{\rho_{12m}^{-}} \right\}-z_{23\eta}^{-^{'}}\frac{1+\mu_{eff}\rho_{23\eta}^{-^{'}}}{\left( \rho_{23\eta}^{-^{'}} \right)^{3}}\left\{ \frac{exp\left[ -\mu_{eff}\left( \rho_{12m}^{+}+\rho_{23\eta}^{-^{'}} \right) \right]}{\rho_{12m}^{+}}-\frac{exp\left[ -\mu_{eff}\left( \rho_{12m}^{-}+\rho_{23\eta}^{-^{'}} \right) \right]}{\rho_{12m}^{-}} \right\} \right) (4)$$

Where, ${R_{i}}^{0}\left( \rho_{j} \right)$ indicates reflectance of homogeneous medium concerning the channel and $\Delta R^{a}\left( \rho_{j},T_{s},i \right)$ refers to reflectance due to change in absorption in the existence of i^th^ inclusion at $\vec{r_{i}}$ and $\rho_{j}$ is the distance between the corresponding source (S_x_) and detector (D_x_). The index “i” represents the index of synthetic hemodynamic inside medium and “j” refers to the index of the channel. Refer to chapter 7 of [16] for the rest of the parameters in the equation (3) and(4).
