# Supplementary material for: Performance assessment of high-density diffuse optical topography regarding source-detector array topology
Source: PLoS One. 2020 Mar 24;15(3):e0230206. doi: 10.1371/journal.pone.0230206 (PMC7092988; doi:10.1371/journal.pone.0230206)
Supplement: Attachment — Submitted filename: report.pdf [file pone.0230206.s004.pdf]

Author report on a simulation platform for diffuse optical tomography (DOT) adapted for reconstruction of hemodynamic responses. The platform is based on analytical solutions, under the Born approximation, of the diffuse equation for a semi-infinite slab. The work is technically sound and, together with the claimed availability of software and data, will be useful for setting up DOT systems.

At my opinion there are the following points to address and clarify:

1. Reconstruction: what I don't understand is whether the reconstruction is forced at the depth of 15 mm or it is performed in the whole volume. In the first case, the point has to be emphasized and better specified in the text and, at my opinion, the method can't be properly called "tomography". In the second case, as well, it has to be emphasized in the text and, what I expect, is a figure representing slices in the volume at a defined time *Ts* to see the reconstructed depth that, in DOT, is typically underestimated.
2. Noise and Inverse-crime: As far as I've understood, the only noise added is on the optical properties of the *S5* and nearby voxels. This means that the forward model has been generated with well-defined optical properties using the Jacobian matrix, and the inversion has been performed using the same matrix on unnoisy data ΔR. This is typically called an "inverse-crime". I suggest to add noise (Gaussian or Poisson) to the simulated data to avoid this problem.
3. Minors:
    a. the acronym HRF is not specified in the text.
    b. Figure 1 is reported with a very poor resolution, writings are too small.
