# Supplementary material for: Performance assessment of high-density diffuse optical topography regarding source-detector array topology
Source: PLoS One. 2020 Mar 24;15(3):e0230206. doi: 10.1371/journal.pone.0230206 (PMC7092988; doi:10.1371/journal.pone.0230206)
Supplement: Attachment — Submitted filename: Rebutal Letter PAMDMD.DOCX [file pone.0230206.s003.DOCX]

**Rebuttal Letter for Submission number: PONE-D-19-11996**

**Dear Madam/Sir**

A recent manuscript entitled "Modeling and Simulation of Cerebral Hemodynamic Response Extraction in Cross-Wise Arrangement of fNIRS Optodes for Improving Spatial Resolution" has obtained substantial modifications based on the suggestion and comments of the respected reviewers of this journal.

According to the comments, the structure of the manuscript and even its title have been changed. This time the process of writing of the article removes any ambiguity associated with the previous version.

We have changed the title to “Performance Assessment of High-Density Diffuse Optical Tomography Regarding Source-Detector Array Topology” to fit the topic of our research and results.

We have developed an analytical simulation setup to evaluate the performance of SD arrangement on hemodynamic reconstruction. This setup is our main contribution. Besides that, the multi-distance multi-directional (MDMD) SD array is proposed to gain the accuracy of hemodynamic retrieval.

Why we named analytical simulation setup? Since the simulation setup consists of a forward model, the solution of the forward model is required to calculate the sensitivity matrix (Jacobian matrix). There are three main approaches to the solution of the forward model: analytical, finite element method (FEM) and Monte Carlo (MC). Recently in 2019, Adam Eggebrecht and colleagues presented a simulation toolbox that is based on FEM (Eggebrecht & Culver, 2019). However we have obtained the solution of perturbative diffusion equation to estimate the solution of forward model. Compared to general forward solvers like the FEM, the perturbation approach shows a significantly lower computation time and greater simplicity (Martelli, Del Bianco, & Zaccanti, 2005). For this reason, in the first step of our research, the analytical method was chosen.

Besides the help of the simulation setup and statistic, we have evolved into a conclusion. We have observed proper hemodynamic reconstruction using the MDMD arrangement. Also, a correlation is found between the number of unique elements in the Jacobian matrix and the accuracy of the cerebral hemodynamic reconstruction.

Finally, the developed simulation setup can be used for performance assessment of high-density diffuse optical tomography (HD-DOT) (Eggebrecht et al., 2014).

The answers to the referee's comments are in two ways. In the first step (first and second section), we have outlined the common concern among all referees, and then we have discussed the concerns and addressed the solution. Then the line by line comments of each reviewer is answered in detail (Third section).

1. **Comparison**

We have compared the result of different SD arrangement in hemodynamic reconstruction. MDMD performance is compared with HD-DOT. The results of Fig.7, Fig.8, Fig.9, and Table 1 represents the comparison.

The depth sensitivity of the analytical forward model (Sassaroli, Martelli, & Fantini, 2006) is compared with the results of MC on Colin 27 brain template (Strangman, Li, & Zhang, 2013). Fig.10 and Fig. 11 represents the comparison in the discussion section

2. **Data Availability**

The simulation codes and data are available from the corresponding author upon a reasonable request.

3. **Answer to applicable suggestions and comments to new version**

The answer to line by line comments of reviewers:

First Reviewer:

1. Comment: Introduction: the references are not consistent with the text. They are good references, but are not cited in the best place. For example, ref 3 refers to EEG but not to the hemodynamic activity and so on. Also ref 7-14 are not adequate: if you tell us something about CW- you have not to cite TD-NIRS, and if you want to explain CW in general, you have not to cite method for motion artifact correction. Please, choose them better. Ref. 5 is not cited.

Answer: Thanks for the comments. We have changed the introduction section. We have added and removed some related works.

2. Comment: The whole paper is to edit from the English point of view. Furthermore, inside the same paragraph the same concept is often repeat in different points, these increase the number of sentence and make confusion.

Answer: Thank you for your valuable comment. The manuscript edited several times to make the text fluent, and we have removed those repeated points.

3. Comment: SOE: it not completely clear. Are you sure that is the best way to represents your results? If yes, please clarify its meaning.

Answer: Thanks for the comment, no it is not the best way to compare the synthetic and reconstructed results. We have changed the cost function to the correlation coefficient. The correlation coefficient is a widely used function to find out the similarity between two signals.

4. Comment: The discussion paragraph is just a list (again) of the things you did in the paper rather than a discussion. This section need an improvement. It is clear what you have done and how but not your results and, in particular, why your optodes arrangement is better respect to others. Please look for similar works in literature and make comparison. In my opinion this is the major point you have to address.

Answer: We have compared the result of different SD arrangement in hemodynamic reconstruction. MDMD performance is compared with HD-DOT. The results of Fig.7, Fig.8, Fig.9, and Table 1 represents the comparison.

Second Reviewer:

1. Comment: First of all, authors claim that the data is available within manuscript and available files. However, I can't find it. What I see is that there were no experiments and only synthetically generated data was used. How exactly was it generated is also not clear.

Answer: Thanks for the comment. The primary concern of all reviewers was that the data is not available. However, we have put all the formulas in the manuscript, and also we had mentioned in the submission process (Cover Letter) that even the Matlab codes would be available upon the request. The synthetic data is generated in section 2.3, please refer to this section for further explanation.

2. Comment: Another issue is that authors claim that the proposed approach is unique and the suggested approach will help fNIRS researchers to select the optimum number of channels and the proper location of the source and detectors. Although they claim it, they haven't shown any analysis of how to optimize the number of channels. How was it optimized, what variable was optimized based on what cost function and in presence of what constrains. These details are missing.

Answer: Of course, the modeling approach and simulation is unique. The number of channels can be optimized based on spatial resolution and the accuracy of the hemodynamic reconstruction. Spatial resolution means that how many hemodynamic in each voxel we can reconstruct regarding the SD arrangement and number of channels. The cost function is correlation between synthetic and reconstructed data. I refer you to Fig.1 and Fig.2, which represents the simulation setup and different SD arrangement. For more information please refer to simulation section.

3. Comment: What actually is the standard spatial resolution of fNIRS?

Answer: Since in the new version of the manuscript we are not stressing on spatial resolution anymore, for this reason, we refer you to this review article (Wheelock, Culver, & Eggebrecht, 2019)

4. Comment: In the absence of a benchmark study, this all analysis is pointless because almost every fNIRS study claims different spatial resolution of different devices. I recommend authors to benchmark a latest paper that attempts to improve spatial resolution of fNIRS and compare your results which that paper. More importantly, data should be acquired using an fNIRs equipment for a fair comparison instead of using synthetically generated data which is pointless because spatial resolution is largely dependent of equipment itself.

Answer: Thanks for the comments. We have added benchmark study (HD-DOT (Eggebrecht et al., 2014)). Benchmark study and proposed MDMD are compared in Table 1 and simulation figures. Since Both arrangements are simulated in the same simulation setup, it would be a fair comparison.

Third Reviewer:

1. Comment: In the manuscript the authors report implementation of a simulation setup for the evaluation of performances of a diffuse optical tomographic approach to fNIRS.
   The paper is decently written, and the rationale and procedures are well described.
   However, i have some concerns to be addressed prior publication
   Throughout the paper it seems that the procedure presented is completely novel. However, the approach of cross-wise arrangement of high-density optodes is well known in the diffuse optical imaging field and it is labelled as Diffuse Optical Tomography (DOT). Some more credit should be given to previous work. (e.g., Boas, D. A., Brooks, D. H., Miller, E. L., DiMarzio, C. A., Kilmer, M., Gaudette, R. J., & Zhang, Q. (2001). Imaging the body with diffuse optical tomography. IEEE signal processing magazine, 18(6), 57-75.
   Eggebrecht, A. T., Ferradal, S. L., Robichaux-Viehoever, A., Hassanpour, M. S., Dehghani, H., Snyder, A. Z., ... & Culver, J. P. (2014). Mapping distributed brain function and networks with diffuse optical tomography. Nature photonics, 8(6), 448.)

Answer: Thanks for the comments. We have changed the introduction section. We have added those related works. Also, we have compared our results with previous work.

2. Comment: In fact, instead of stressing the cross-wise arrangement of optodes as the research novelty (remove sentences such as the one in line 301, To best of our knowledge..), i would stress in the manuscript the implementation of the simulation (including both spatial and temporal characteristics
   of fNIRS measurements) as a possible tool for the assessment of the performances of fNIRS DOT probably moving figure 13 at the start of the method section

Answer: Thank you for your valuable comment. Your comments were a great help to improve the manuscript. We have moved figure 13 in previous version to Fig.1.

3. Comment: There are too many figures, I would remove figure 6,7 and 8 and leave only figure 9

Answer: Thanks for the comment. Those figures are removed.

4. Comment: I would express hemoglobin concentrations in microM and not nM in figures

Answer: Thanks for the comment. It is done. All the result are in microM.

5. Comment: I would go through one more round of English revision

Answer: Thanks for the comment. It has been reviewed multiple times.

**References**

Eggebrecht, A. T., & Culver, J. P. (2019). NeuroDOT: an extensible Matlab toolbox for streamlined optical functional mapping. *Diffuse Optical Spectroscopy and Imaging VII*, *11074*, 110740M.

Eggebrecht, A. T., Ferradal, S. L., Robichaux-Viehoever, A., Hassanpour, M. S., Dehghani, H., Snyder, A. Z., … Culver, J. P. (2014). Mapping distributed brain function and networks with diffuse optical tomography. *Nature Photonics*, *8*(6), 448–454. https://doi.org/10.1038/nphoton.2014.107

Martelli, F., Del Bianco, S., & Zaccanti, G. (2005). Perturbation model for light propagation through diffusive layered media. *Physics in Medicine & Biology*, *50*(9), 2159.

Sassaroli, A., Martelli, F., & Fantini, S. (2006). *Perturbation theory for the diffusion equation by*. *23*(9), 2119–2131.

Strangman, G. E., Li, Z., & Zhang, Q. (2013). *Depth Sensitivity and Source-Detector Separations for Near Infrared Spectroscopy Based on the Colin27 Brain Template*. *8*(8). https://doi.org/10.1371/journal.pone.0066319

Wheelock, M. D., Culver, J. P., & Eggebrecht, A. T. (2019). High-density diffuse optical tomography for imaging human brain function. *Review of Scientific Instruments*, *90*(5). https://doi.org/10.1063/1.5086809
